# Anatomical limits on interaural time differences: an ecological perspective

**DOI:** 10.3389/fnins.2014.00034

**Published:** 2014-02-28

**Authors:** William M. Hartmann, Eric J. Macaulay

**Affiliations:** Psychoacoustics Laboratory, Department of Physics and Astronomy, Michigan State UniversityEast Lansing, MI, USA

**Keywords:** brainstem, evolution, binaural, sound localization, interaural time difference, spherical head model, rotation-azimuth transform

## Abstract

Human listeners, and other animals too, use interaural time differences (ITD) to localize sounds. If the sounds are pure tones, a simple frequency factor relates the ITD to the interaural phase difference (IPD), for which there are known iso-IPD boundaries, 90°, 180°… defining regions of spatial perception. In this article, iso-IPD boundaries for humans are translated into azimuths using a spherical head model (SHM), and the calculations are checked by free-field measurements. The translated boundaries provide quantitative tests of an ecological interpretation for the dramatic onset of ITD insensitivity at high frequencies. According to this interpretation, the insensitivity serves as a defense against misinformation and can be attributed to limits on binaural processing in the brainstem. Calculations show that the ecological explanation passes the tests only if the binaural brainstem properties evolved or developed consistent with heads that are 50% smaller than current adult heads. Measurements on more realistic head shapes relax that requirement only slightly. The problem posed by the discrepancy between the current head size and a smaller, ideal head size was apparently solved by the evolution or development of central processes that discount large IPDs in favor of interaural level differences. The latter become more important with increasing head size.

## 1. Introduction

**Figure 1:**
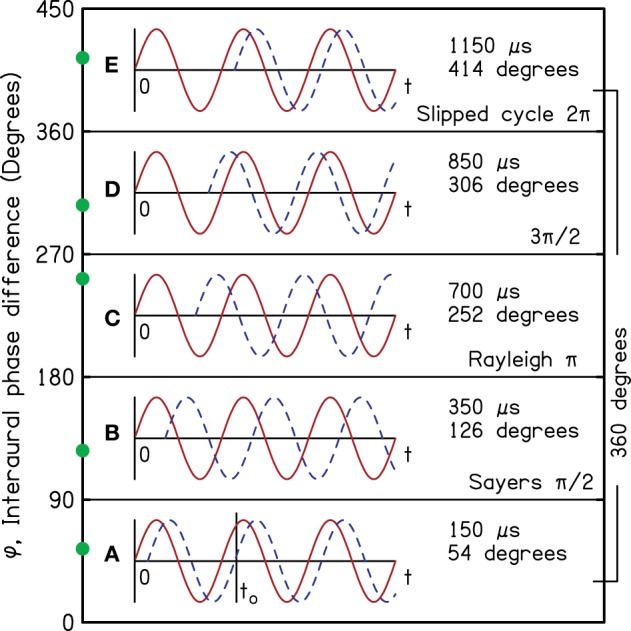
**Tones in the right ear (red) and left ear (blue and dashed) as functions of time and with particular interaural phase differences (IPD) as indicated on the vertical axis to illustrate different regions of IPD**. The boundaries between regions, separated by 90°, are logically and perceptually important in sound localization.

More than 100 years ago, Lord Rayleigh pointed out that human listeners can make use of interaural time differences (ITD) to localize pure tones (Strutt, 1907). An example is illustrated by the functions in Figure 1, which represent the pressures at the two ears for a 1000-Hz tone. Here, the source of the tone is on the listener's right side so that the waveform in the right ear (red) starts before the waveform in the left (blue and dashed). As shown in region A, the ongoing wave in the right ear continues to lead the ongoing wave in the left. For instance, the positive-going zero crossing at time *t*_*o*_ in the left ear is preceded by a similar crossing in the right.

### 1.1. The interaural phase problem

Rayleigh was quick to point out that there are practical limits to the utility of the ITD. When the azimuth increases enough that the interaural phase difference (IPD) becomes equal to 180°, the ongoing information from the ITD becomes totally ambiguous. As the azimuth increases further, and the IPD exceeds 180° (regions C and D), the ITD points to images with azimuths opposite to the actual source azimuth. Headphone experiments by Bernstein and Trahiotis (1985) have revealed just this kind of ambiguity. Thus, there is a 180° IPD limit on useful ITD cues. Region D is especially misleading—even dangerous. Although the source continues to be on the listener's right, the ongoing waveform indicates that the source is on the left—just as surely as it pointed to a source on the right in region A. In free-field listening, this misleading ongoing information actually dominates the (correct) onset information (Hartmann and Rakerd, 1989).

Sayers (1964) reported experiments indicating another IPD boundary of interest. As the ITD increases such that the IPD exceeds about 90° (region B), further increases in ITD cause the image to move back toward the midline. Also, in region B listeners sometimes lateralize images on the wrong side of the head. Yost (1981) similarly found frequent wrong-side lateralization in region B, and Elpern and Naughton (1964) showed that the maximum sensation of lateralization occurs for IPD = 90°. Thus, there is a 90° IPD limit on useful directional information from *changes* in the ITD, and the regions of ITD information are logically represented by IPD boundaries separated by 90° as shown in Figure 1.

Region E shows a confusion of yet another sort. Here, the ongoing waveforms are identical to those in region A, but the ITD in region E is larger by a full period of the tone (1000 μs). The same ongoing waveform corresponds to two different ITDs, indicating two different characteristic delays of the same sign, potentially associated with two different locations on the same side of the head.

It has been proposed that the IPD confusions noted here have been ameliorated by a binaural system that becomes insensitive to ITDs at high frequency. This idea will be called the “ecological interpretation,” and the rest of this article will study its plausibility and possible modifications to it.

### 1.2. Transformations

**Figure 2:**
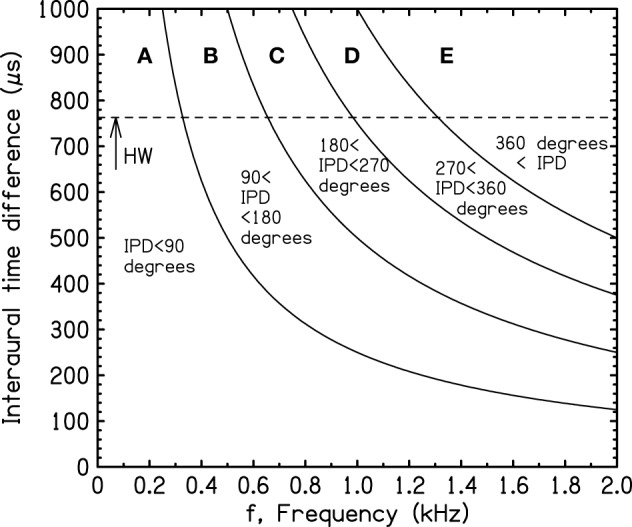
**Transformation of the iso-IPD boundaries in Figure 1 to a scale of frequency and interaural time difference (ITD)**. HW indicates the largest possible ITD for the average human head in free field.

Because the IPD is the product of the ITD and the frequency of the tone, the IPD boundaries of Figure 1 can be translated to ITD and frequency, as shown in Figure 2. These boundaries will be called “iso-IPD contours” or simply “IPD contours” or “IPD boundaries.” The dashed horizontal line (HW) indicates the largest ITD that can be caused by the typical human head for sound sources in free field, sometimes called the Hornbostel–Wertheimer constant (von Hornbostel and Wertheimer, 1920). Figure 2 shows it as the low-frequency limit of the head diffraction formula ITD = (3*a*/*v*) sin(90°) = 763μs. Here *a* (8.75 cm) is the radius of the typical human head (Hartley and Fry, 1921; Algazi et al., 2001), and *v* (34,400 cm/s), is the speed of sound in room-temperature air.

**Figure 3:**
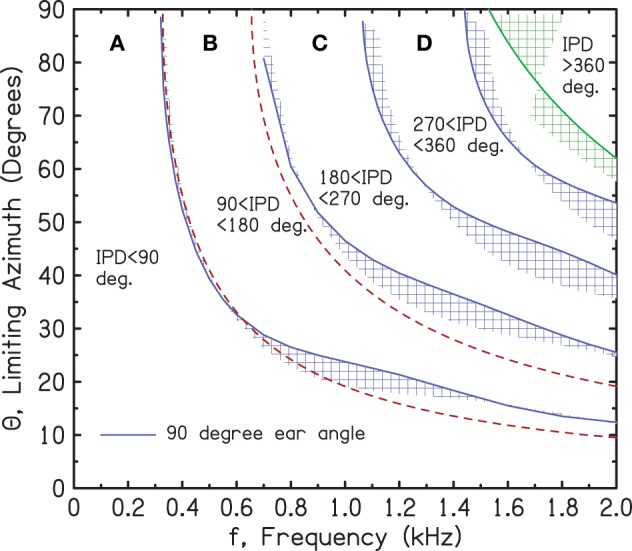
**Transforming the ITD axis in Figure 2 to an azimuthal axis using the spherical head diffraction model**. The blue shaded regions are bounded by ear angles of 90° (solid blue line) and 110°. The green shaded region similarly shows the Woodworth model. The red dashed curves show the low-frequency limit of the spherical head model for IPDs of 90° and 180°.

Figure 2 shows that the iso-IPD contours, such as the 90° or 180° boundaries, are not important if the ITD is small or the frequency is low. Small ITDs occur in the real world when the azimuth of the source is small. Large ITDs, and large IPDs, occur when the source is off to the side of the listener. A representation in terms of source azimuth can be obtained by transforming the ITD axis in Figure 2 to a scale of source azimuth, as shown in Figure 3.

## 2. Spherical head model

The shaded regions in Figure 3 are transformations to an azimuthal scale using a spherical head model (SHM). The iso-IPD contours separating the regions in Figure 2 have become thin regions corresponding to different locations of the ears on the head.

### 2.1. Spherical head calculations

The calculations for Figure 3 were based on an exact mathematical treatment of the scattering of waves by a rigid sphere. Solutions to this scattering problem for plane wave incidence (infinite source distance) go back as far as Rayleigh (1896). A modern solution, which is a series of Legendre polynomials with frequency-dependent, complex spherical functions as coefficients, was given by Rschevkin (1963) and applied to interaural differences for a spherical head by Kuhn (1977). The spherical head calculation was generalized to finite source distance by Rabinowitz et al. (1993) and Duda and Martens (1998). In the limit of infinite source distance, the finite-distance solution reduces to Kuhn's result. Our Figure 3 used the finite-distance solution with a source distance of 2 m to match experiment. However, there is actually very little difference between ITDs computed for a source at 2 m and a source at infinity. (The interaural level difference is much more sensitive to source distance.) The spherical head solution captures the important frequency dependence of the ITD that is also characteristic of human heads. The frequency dependence of the ITD for different azimuths, as plotted by Constan and Hartmann (2003) (their Figure 1), shows a significant drop in ITD between 400 and 2000 Hz.

The low-frequency limit, (3*a*/*v*) sin(θ) generally underestimates the ITD at low frequency. For instance, Kuhn (1977) found that in order to match low-frequency KEMAR ITDs, it was necessary to increase the head radius from *a* = 8.75 to 9.3 cm. Kuhn tentatively attributed the apparent extra size to the pinnae, which would be indistinguishable from the bulk of the head when viewed with wavelengths corresponding to low frequencies. Fortunately, all the frequencies of interest in the current article are greater than 600 Hz, and in this range, the SHM ITD agrees better with measurements on human listeners. The high-frequency limit of the SHM is the creeping wave solution known as the Woodworth model (Woodworth, 1938). In this limit ITDs are smaller than in the low-frequency limit, with the decrease depending on the azimuth. For small azimuths, the high-frequency limiting ITD is 33% smaller than the low-frequency limit. At the other extreme, an azimuth of 90°, the high-frequency ITD is only 14% smaller.

The shaded contours in Figure 3 arise from a range of assumptions about the angle of the listener's ears with respect to the forward direction. The boundaries indicated with solid blue lines correspond to an ear angle of 90°; the other edges of the shaded regions correspond to 110°. Thus, the contours are centered on an ear angle of 100°, as suggested by Blauert (1997) and used by Duda and Martens (1998) and by Treeby et al. (2007). For comparison, we note that Hartley and Fry (1921) suggested that the human ear is 97.5°.

The red, dashed lines represent the low-frequency (*f*) limit of the azimuth (Θ) for a spherical head with radius *a*: Θ = arcsin[*v*/(6*fa*)] for the 180° IPD limit and Θ = arcsin[*v*/(12*fa*)] for the 90° IPD limit.

As expected, the low-frequency limit agrees with the exact formula for a 90° ear angle near 400 Hz and departs from the exact formula as the frequency increases. The green, shaded region at high frequency shows the 360° IPD contour from the Woodworth model, which is only valid at high frequency. The calculations for ear angles between 90° and 110° were made using formulas for the Woodworth model from Aaronson and Hartmann (2014). This latter article shows that unless the frequency is very high, the Woodworth formula underestimates the ITD. That is why, for every frequency, an especially large azimuth is required to produce a given IPD—in this case, an IPD of 360°.

### 2.2. Spherical head array measurements

The spherical head calculations in Figure 3 were tested against measurements of frequency and azimuth that targeted IPDs of interest. Measurements were made in an anechoic room (7.7 × 6.4 × 3.6 m) (IAC 107840) using an array of 13 loudspeaker sources (Minimus 3.5) spaced by 7.5° and located 2 m away from a binaural receiver. The array was a single quadrant (0–90°) to the right of the receiver. The receiver was a rigid spherical shell (Shapemaster, Ogden, IL) with a radius of 8.75 cm made of 6-mm PETG (glycol-modified polyethylene terephthalate) and mounted on a microphone stand 117 cm off the wire grid floor, the same height as the array sources. The forward direction of the sphere was defined by a laser beam through the center of the sphere. Two small holes were drilled at 90° from the forward direction to accommodate the ends of the probe tubes (0.95 mm O.D.) of Etymotic ER-7c probe microphones. (Etymotic Research, Elkgrove Village, IL). Therefore, the simulated ear angles were 90°. Signals from the microphones were first amplified with the associated probe-tube-compensating Etymotic preamplifier, and then given another 40 dB of gain before conversion to digital form by a DD1 two-channel 16-bit analog-to-digital converter (Tucker-Davis Technologies, Alachua, FL). Because the frequency of the signal was exactly known, it was possible to use matched filtering to process half-second samples of the digitized signals and to extract precise IPDs.

**Figure 4:**
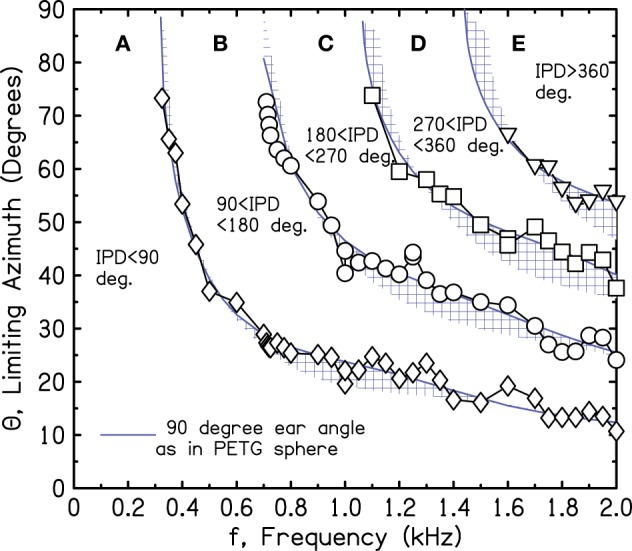
**Measured values of frequency and azimuth that lead to IPDs of 90°, 180°, 270°, and 360° (diamonds, circles, squares, triangles, respectively) for a perfect sphere**. Values were interpolated from measurements using a source array in one quadrant.

Estimates for the target IPD boundaries of 90°, 180°, 270°, and 360° are shown in Figure 4. They were determined by setting the frequency to successive values and measuring IPDs for the 13 sources. Then, source azimuths for the target IPD boundaries were interpolated from the measured IPDs. The interpolation procedure required the assumption that the IPD-azimuth relationship was smooth and locally linear. Figure 4 shows that the interpolated azimuths agree reasonably well with the solid lines at the tops of the shaded regions, as expected for a 90° ear angle.

### 2.3. Spherical head rotation measurements

**Figure 5:**
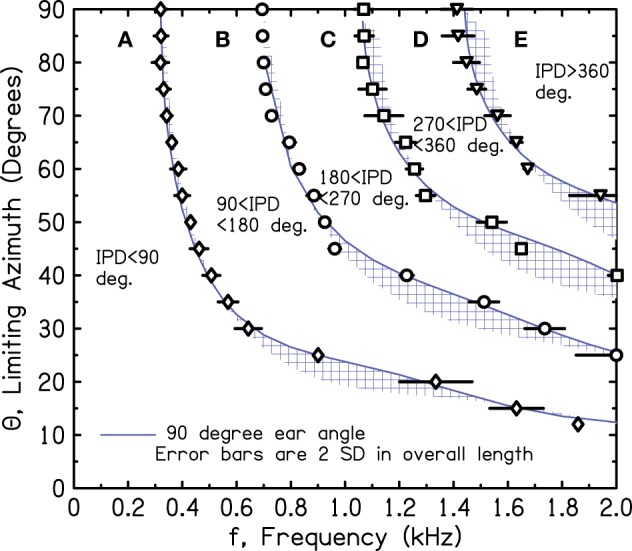
**Measured values of frequency and azimuth that lead to IPDs of 90°, 180°, 270°, and 360° for a perfect sphere**. Values were measured in four quadrants using a single source and rotating the sphere. The average of the four is shown together with an error bar two standard deviations in overall length.

Because of our concern with the interpolated array measurements over 7.5° and with inadvertent scattering from the array structure itself, we repeated the IPD boundary measurements on the sphere using only a single loudspeaker source, 3 m from the sphere, in the anechoic room. The different source azimuths were obtained by rotating the sphere with its microphone stand using a calibrated rotating table on the wire grid floor. To make measurements, the sphere was rotated to a desired azimuth, and the frequency was varied to hit a targeted IPD. Thus, the procedure involved no interpolation. Unfortunately, the microphone stand could not be made perfectly vertical. To compensate, the measurements were made four times, rotating through 90° in all four quadrants with the expectation that the effect of the wobble would be mostly canceled in the average. The averages with standard deviations over the four rotations are shown in Figure 5. Again, the symbols lie close to the solid line for the 90° ear angle. In the end, the good agreement between the calculations and the measurements from both the array and the rotated head suggest good correspondence between the SHM and free-field reality for the IPDs of interest.

Figures 2–5 show that when the frequency is low, the IPD is within the most useful region, namely region A—0° to 90°. So long as the frequency is less than a critical value where the 90° iso-IPD contour intersects the top axis, region A applies for all azimuths, 0–90°. The SHM and our measurements agree that this critical frequency is well approximated by the low-frequency limit of the model, *v*/(12*a*) or 328 Hz. Similarly, the IPD completely avoids the ambiguous 180° boundary and region C only if the frequency is less than 328 × 2 or 655 Hz. As the frequency increases beyond this value, the ambiguity and the misinformation provided by the ITD start to occur at ever smaller values of the azimuth. An important conclusion to be drawn from Figures 2–5 is that both the 180° and the 90° iso-IPD boundaries are exceeded for tones with frequencies that are *not particularly high* and for azimuths that are *not particularly large*. The boundaries would appear to be real problems for the use of ITD cues in real-world sound localization.

## 3. Human ITD sensitivity

Because ITD information becomes increasingly misleading as the frequencies and azimuths increase, there would be survival value in a binaural system that becomes insensitive to ITD at moderately high frequency. Such a system would defend its owner from dangerous localization cues that could lead to mislocalization. In fact, there is unequivocal evidence that fine-structure ITD sensitivity disappears at about 1500 Hz. The upper limit of ITD sensitivity was explored by Zwislocki and Feldman (1956) and by Klumpp and Eady (1956), who found an upper limit of 1300 Hz. Mills (1958) found a limit of 1400 Hz, and Nordmark (1976) found 1430 Hz.

**Figure 6:**
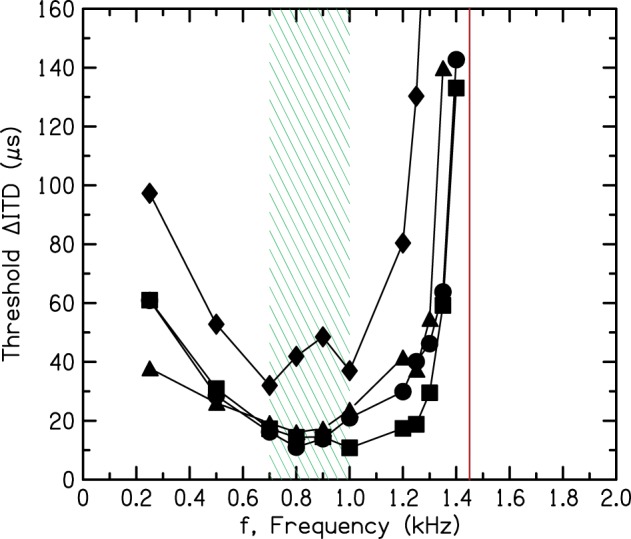
**Threshold interaural time differences as a function of frequency for four listeners measured by Brughera et al. (2013)**. The shaded rectangle indicates the frequency region of greatest sensitivity. The vertical solid line shows the brick wall.

The most detailed exploration of the frequency dependence of ITD sensitivity was recently made by Brughera et al. (2013), paying particular attention to the high-frequency limit. The procedures in that work were approved by the Michigan State University institutional review board, and informed consent was obtained from all subjects. That exploration used a two-interval forced-choice task in which a tone led in one ear by the ITD on the first interval and led in the other ear by the ITD on the second. The difference between the two intervals, ΔITD (twice the ITD on each interval) is plotted in Figure 6. The thresholds in Figure 6 show a broad minimum between 700 and 1000 Hz indicating the frequency region of greatest sensitivity. They show a sharp rise above 1200 Hz. Brughera et al. found that some listeners were sensitive to the ITD at 1400 Hz, but all listeners found it impossible to detect the ITD at 1450 Hz, in good agreement with Nordmark.

The shaded rectangle in Figure 6 between 700 and 1000 Hz indicates the frequency range of greatest sensitivity to ITD. The vertical line in Figure 6 at 1450 Hz indicates the upper limit. Because we are unaware of any experiment indicating ITD sensitivity for a tone with a frequency greater than 1450 Hz, the rest of this article will refer to the boundary at 1450 Hz as the “brick wall.” It is striking that the frequency difference between the top of the region of greatest sensitivity and the brick wall is considerably less than an octave. It is an unusually sharp transition.

The loss of ITD sensitivity for sine tones above 1450 Hz is consistent with other binaural phenomena, such as binaural beats, which indicate a loss of interaural phase sensitivity near this frequency (Perrott and Nelson, 1969). Although the binaural masking level difference (MLD) is a more complicated effect, there is evidence of a similar limit in a dozen experiments cited by Durlach (1972), where the MLD as a function of frequency shows a discontinuity in slope near 1500 Hz (Durlach Figure 4).

The loss of phase sensitivity at the brick wall appears to be specifically a binaural phenomenon. There is good reason to believe that phase locking is maintained in the human auditory system for considerably higher frequencies. A low estimate for the loss of phase locking (between 2 and 3 kHz) comes from mistuned harmonic detection experiments (Hartmann et al., 1990). A high estimate (8 kHz) comes from frequency difference limen experiments (Moore and Ernst, 2012). Intermediate estimates (4–5 kHz) come from musical pitch experiments (e.g., Oxenham et al., 2011) or from assuming that phase locking in humans is similar to the auditory nerve of cat (Johnson, 1980). Apparently there is an especially low limit for the human binaural system. But although the lowpass character must follow the initial stage of binaural interaction, it is not certain where it originates. The neural modeling by Brughera et al. (2013), based on cat and gerbil physiology, identified the superior olive complex in the brainstem as the origin of the low limit. Whether the limit occurs in the superior olive or in the inferior colliculus, it is not unreasonable to focus on the brainstem and to conjecture that the limit represents an evolutionary adaptation of the brainstem to ITD values of negative utility as seen in Figures 2–5.

## 4. The ecological interpretation

An ecological interpretation for the high-frequency limits of ITD sensitivity has often been proposed. Rayleigh (Strutt, 1907) argued that it was unlikely that listeners could localize sounds based only on ITD when the frequency was much above 512 Hz because the maximum delay across the head (about 800 μs) would lead to an IPD close to 180°. In 1909, Rayleigh (Strutt, 1909) also remarked on the 90° IPD boundary, leading to an even lower estimate for the maximum frequency for useable ITD. Yost and Hafter (1987) noted that delaying a 1666-Hz tone by a head width would be equivalent to no delay at all (region E). The 2005 review of binaural hearing by Stern et al. (2005) similarly suggested that the upper limit of ITD utility should be set by the size of the head. Moore's introduction to human hearing (1997) also noted the correspondence between the ambiguity of the ITD cue and the distance between the ears. Taking a somewhat different direction, Blauert (1997) argued that the head size establishes an upper limit of about 630 μs on useful ITDs. Schnupp et al. (2011) argued similarly, applying the same principle to all animals. Carlile (1996) noted that the only unambiguous tones are those with wavelength less than twice the head radius. Calculations by Harper and McAlpine (2004) showed that the optimum array for coding of cross-correlation in IPD-frequency space is mainly a function of an animal's head size.

**Figure 7:**
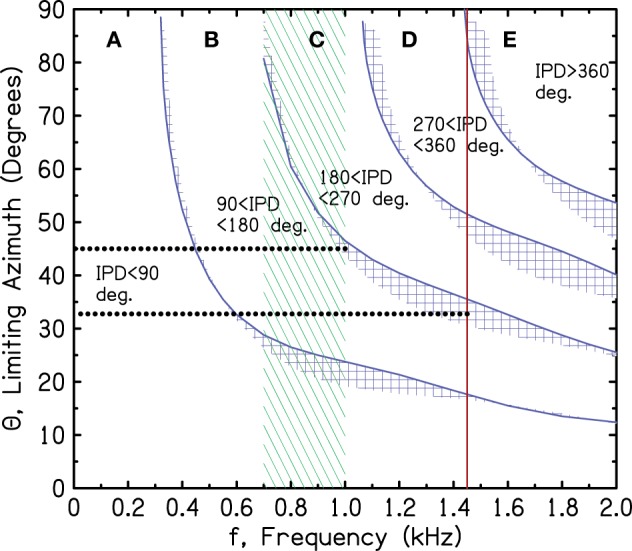
**Sensitivity regions from Figure 6 together with model boundaries for the IPD regions from Figure 3**. The dotted lines refer to the argument in the text against the ecological interpretation given present-day human head sizes.

As shown in Figures 2–5, the azimuths for the boundaries IPD = 90° and 180° are rapidly varying functions of frequency in the large azimuth regime. As shown in Figure 6, the ITD sensitivity also has a rapid frequency dependence. According to the ecological interpretation (EI), these regions of changing sensitivity ought to be sensibly related. Figure 7 repeats the spherical head regions from Figure 3, and also repeats the region of greatest ITD sensitivity and the brick wall from Figure 6. Figure 7 shows that the relationship is far from sensible.

As shown by the dotted lines in Figure 7, for the 180° boundary, the EI would assert that the binaural system has become insensitive to 1450-Hz tones because the IPD exceeds 180°, leading to wrong-sided images, whenever the azimuth is greater than 33°. By contrast, the binaural system has remained highly sensitive to 1000-Hz tones because they are more reliable. They lead to wrong-sided images only when the azimuth is greater than 45°. The problem with this picture is that the difference of only 12° of azimuth is hardly adequate motivation for a system to develop such a sharply tuned frequency response as the human binaural ITD system evidently has.

The corresponding analysis for the 90° iso-IPD contour (not shown in the figure) is even more disappointing. According to the EI, the binaural system rejects ITD information from a 1450-Hz tone because this tone leads to perceived images that move in directions opposite to reality when the azimuth is greater than 14°. By contrast, the binaural system maintains sensitivity to ITD information at 1000 Hz because it leads to misleading directional information only when the azimuth is greater than 24°. Again, the difference of only 10° seems to be a poor reason to evolve an ITD with a sharp frequency cutoff. Given the poor correspondence between the IPD boundaries and the limits of ITD sensitivity, one is tempted to abandon the ecological interpretation, at least in the quantitative detail presented here. Perhaps evolutionary pressures are actually responsible for the anomalously low cutoff frequency of ITD sensitivity, but then evolution stopped too soon and didn't get the cutoff quite low enough.

**Figure 8:**
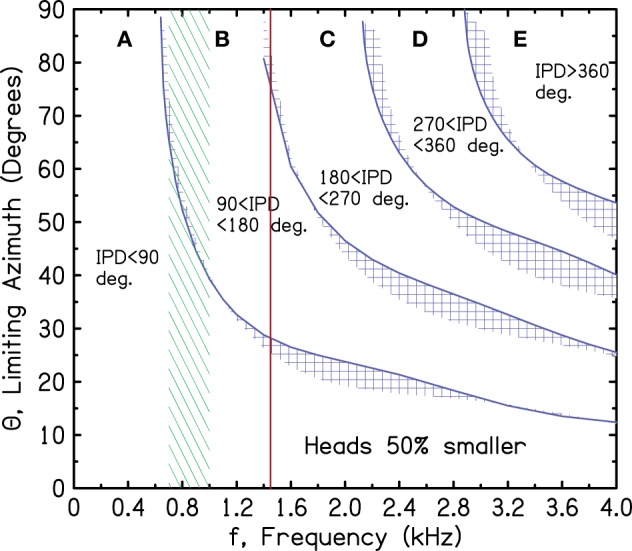
**Same as Figure 7 for a head diameter that is half as large as present-day human heads**.

There is an alternative ecological theory, however, that leads to quantitatively good correspondence. The theory assumes that while the brainstem was evolving, and the medial superior olive and projections to it were developing, the head size was considerably smaller than the current human head. Figure 8 is a repeat of Figure 7 except that it makes the *small-head hypothesis*, assuming that the head is 50% smaller than our present-day human heads—a factor of 2 in diameter.

In Figure 8 the upper limit of ITD sensitivity at 1450 Hz essentially eliminates the confusing ITDs in regions C, D, and E from contributing to sound localization. Only tones with an IPD less than the 180° iso-IPD contour can contribute. In another benefit, the most sensitive region between 700 and 1000 Hz extends to source azimuths as large as 60°. For the 90° iso-IPD contour, ITD information for 1450-Hz tones would be rejected because it leads to an incorrect sense of motion when the azimuth is greater than 27°. The confusing 90° iso-IPD contour does not enter the region of greatest ITD sensitivity until the azimuth has reached 40° (up from 23°). Therefore, a binaural system that developed to optimize ITD coding for a head diameter that is half as large appears to make sense acoustically. It makes some sense in evolutionary terms too because the brainstem is old brain, whereas the head expanded over very recent times to accommodate the neocortex.

A factor of two in diameter, however, may be extreme. Over the past 3.2 million years the brain size has expanded by a factor of 3 (Lynn, 1990). The cube root of 3 is 1.44 suggesting a head diameter that was 30% smaller than present day. Making the head diameter 30% smaller (not shown in the figures) confers some advantages. Then the brick wall at 1450 Hz totally eliminates the most dangerous region, region D, for all azimuths.

The small-head hypothesis carries with it the assumption that the binaural properties of the brainstem have not greatly changed since the origin of homo with rapidly growing heads. That assumption can certainly be challenged because there is evidence that the binaural system changes—even in a single individual, even over a brief time. Evidence for changeable binaural processing is found in studies of development and plasticity. Experiments by Shinn-Cunningham et al. (1998), in which human auditory spatial maps were altered by feedback, or experiments by Hofman et al. (1998), where maps were altered by plugging one ear, show at least partial adaptation to new conditions. It is possible though that short-term accommodations such as these are entirely the result of cortical plasticity, revealing nothing about the brainstem. Concerning the brainstem itself, auditory brainstem response (ABR) experiments, as described in the review by Tzounopoulos and Kraus (2009), indicate plasticity in the brainstem that is both synaptic and intrinsic. The intrinsic plasticity shows changes at a fundamental biochemical level—a likely origin for the ITD brick wall. If brainstem plasticity appears on the time scale of a brief experiment or the development of a single individual, it seems unlikely that the binaural system would be resistant to ecological pressures for a few million years.

In contrast to the plasticity argument above, we conjecture that the binaural system, once adjusted for the ITDs available with small heads, did not change over evolutionary times because evolution found an alternative way to solve the problem of misleading ITDs, namely by using interaural level differences (ILD), which grew to be substantial as the head grew.

Calculations within the SHM show that the ILD is adequate to solve the problem in regions B, C, and D of Figure 7. Along the 90° iso-IPD contour (limit of region B), the ILD is greater than 2 dB except for the lowest frequencies, below 500 Hz. Even at the lowest frequencies the ILD is greater than 2 dB if the source is closer than 2 m. Along the 180° iso-IPD contour (limit of region C), the ILD is always greater than 3.5 dB and usually is much larger. ILDs of these magnitudes are adequate for human listeners to localize on the correct side of the head especially because the ITD cues are weak in these regions. Region D is somewhat more problematical. There, misleading ITD cues can be strong, and the correct ILDs along the 270° iso-IPD contour from 1100 to 1500 Hz are only slightly larger than along the 180° contour, partly because the relevant azimuths become large enough to involve the acoustical bright spot (Macaulay et al., 2010). Although region D, with strong, but wrong, ITD cues, represents more of a problem than region C, it is possible for the misleading ITD cues in both regions to be overcome at a higher level by a process that discounts ITD cues by contravening ILD cues.

The ILD does not solve the confusion problem in region E, where both the ITD and the ILD point in the same direction, and the ITD points to a secondary azimuth. However, Figure 7 (current head size) shows that region E is perfectly eliminated by the brick wall at 1450 Hz.

## 5. Kemar measurements

The experimental approach to the ecological interpretation using the spherical head (section 2) was consistent with historical approaches from the time of Rayleigh to the present. It probably applies to human heads better than to the other mammals that are frequently studied. It is possible, however, that the properties of real human heads might differ from the (SHM) in some important way with consequences for the theory. To obtain measurements of the IPD boundaries that are more realistic, we used a KEMAR manikin (large ears). As for the perfect sphere, we made two different measurements in the anechoic room, one with the 2-m array of 13 sources and the other with a rotating receiver and a single source. The sources were again at ear height.

Tones of fixed frequency were reproduced by the sources, and were recorded by the Etymotic ER-11 microphones within the KEMAR head and associated electronics. The recordings were again processed by matched filtering to obtain IPDs.

### 5.1. Array measurements

**Figure 9:**
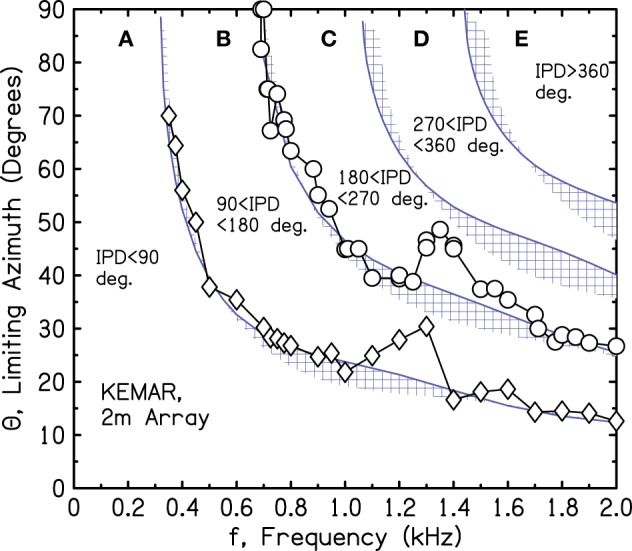
**Measured values of frequency and azimuth that lead to IPDs of 90° and 180° for a KEMAR manikin**. Values were interpolated from measurements using a source array in one quadrant to the right of the manikin.

The source azimuths leading to 90° and 180° IPDs were determined by linear interpolation within the 2-m array for a series of tone frequencies. The results are shown in Figure 9 by circles and diamonds, which follow a smooth descending pattern except for prominent bumps near 1.3 kHz. We noted that a frequency of 1.3 kHz is close to the brick wall.

We suspected that the bumps were due to reflections from the manikin torso, and to test that idea we separated the head from the torso and mounted it on a microphone stand. However, the bumps persisted—somewhat changed in shape but at about the same frequencies. We next questioned the microphone system intrinsic to the KEMAR, and as a check on that system, we replaced it by probe microphones in the KEMAR ear canals (Etymotic ER-7c with associated electronics). The measurements with the alternative system almost perfectly reproduced those made with the KEMAR microphone system, including the bumps.

Because the bumps in the iso-IPD contours were observed in all our KEMAR head configurations and not observed in the array measurements using the perfect sphere, we tentatively concluded that the bumps near 1.3 kHz were caused by diffraction by the KEMAR head itself. However, the interpolated measurements from the array make assumptions about the smoothness of the contours, and those assumptions might not hold for a complicated head structure.

### 5.2. Rotating kemar measurements

To check the measurements made with the array, we used a single loudspeaker 3 m away from the KEMAR, as for the rotated sphere measurements. We obtained different source azimuths by rotating the KEMAR with its mounting pole as an axis. However, unlike the sphere, the axis of rotation did not pass through the center of the head (COH). To relate angles of rotation to source azimuths, we developed the mathematics in Appendix, which solves the problem in principle. The KEMAR has a “+” sign on the top of its cranium and we took that point to be the COH for all measurements. The perpendicular distance from that point to the axis of rotation is 2 cm. As shown in the Appendix, the rotation-azimuth transformation depends on the ratio of this distance to the source distance, in this case a ratio of 2/300. With this value, the formula in the Appendix leads to an angular discrepancy of 0.5°, an error that can be ignored for our purposes.

**Figure 10:**
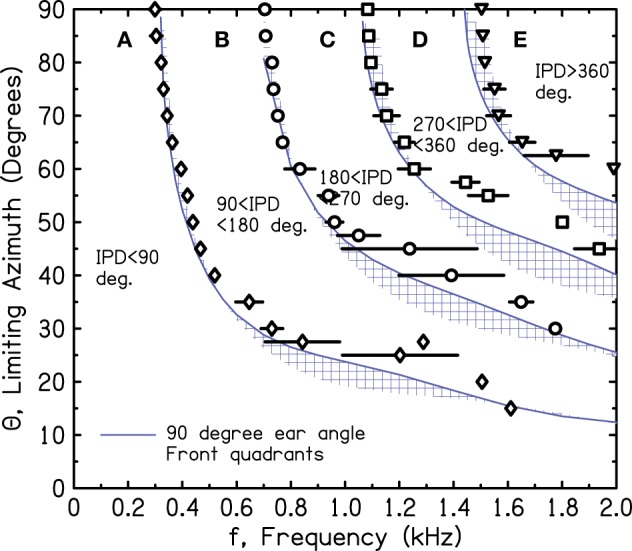
**Measured values of frequency and azimuth for IPDs of 90°, 180°, 270°, and 360° for a rotated KEMAR manikin**. Values were measured in left and right quadrants in *front* of the head using a single source. The average of the two quadrants is shown together with an error bar two standard deviations in overall length. Long error bars indicate regions of non-monotonic IPD.

**Figure 11:**
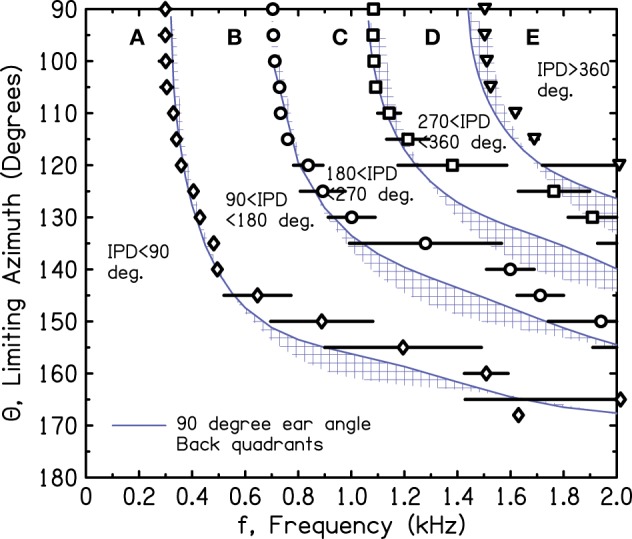
**Measured values of frequency and azimuth for IPDs of 90°, 180°, 270°, and 360° for a rotated KEMAR manikin**. Values were measured in left and right quadrants in *back* of the head using a single source. The average of the two quadrants is shown together with an error bar two standard deviations in overall length. Long error bars indicate regions of non-monotonic IPD.

Figure 10 shows the iso-IPD contours with mean and standard deviation measured across the two frontal quadrants. Figure 11 shows the same for the two back quadrants. Although the details of the plots are not identical to Figure 9, the overall shape is the same, and the bumps for the 90° and 180° iso-IPD boundaries occur at the same frequencies. Figures 10, 11 also show that the bumps occur at higher frequencies for the higher iso-IPD boundaries. The iso-IPD boundary measurements are similar for sources in front of the head (Figure 10) and sources behind the head (Figure 11). Some of the error bars seem rather long, especially as the frequency increases. However, these error bars don't represent actual errors. Instead, they represent regions of frequency and azimuth where the IPDs are not monotonic functions and oscillate around the boundary value. These badly-acting regions became evident as we rotated the head and varied the frequency. It also became evident that the disagreements between Figures 9 and 10 owe much to the failure of the assumptions of smoothness and linearity which limit the accuracy of the interpolated values in Figure 9.

Our measurements have not been able to identify the feature of the head that is responsible for the mid-frequency bumps. The bumps occur at frequencies that are too low to be attributed to detailed anatomical features such as the pinnae. It is possible that they result from the overall elliptical shape of the head. Figures 9–11 show that the effect of the bumps is to push the iso-IPD contours to somewhat higher frequencies and azimuths. Therefore, the useful region A is expanded in azimuth-frequency space. Figures 10, 11 show that the region that is both allowed by the 1450-Hz brick wall and outside the misleading IPD region C is expanded by 5° or 10° of azimuth by the bumps. Alternatively one can observe that the frequency of the 180° IPD boundary for a given azimuth is increased. For instance, for an azimuth of 45° the boundary increases from about 1 to 1.2 kHz, which is in the right direction to agree better with the frequency of the brick wall.

## 6. Discussion

### 6.1. The problem

A central element of the Duplex Theory of sound localization is that ITDs in the fine structure of the sound cease to be informative once the frequency has exceeded a certain limit. The localization error measurements by Stevens and Newman (1936) have been interpreted (even recently) as indicating that the limiting frequency is 3000 Hz. However, 3000 Hz is far too high. The brick wall, which sets an upper limit for any use of ITD fine structure, is lower by a full octave. A limiting value of 1.5 kHz was suggested by Sandel et al. (1955), and this limit approximately agrees with the highest frequency for which ITD sensitivity can be measured (Brughera et al., 2013). The high-frequency limit has frequently been associated with the onset of ambiguities in the IPD caused by the rather large size of the human head. Attributing the high-frequency limit to the head size is the “ecological interpretation” (EI). Because the loss of fine-structure ITD sensitivity near 1.5 kHz is dramatically rapid, it is natural to look for a cause, and the EI provides one. However, to date, arguments for the EI have been quantitatively imprecise. The present article includes model calculations and experiments that make the statement of the EI more quantitative and precise. The calculations and experiments focused especially on critical iso-IPD boundaries where perceptions change. The calculations were all done with the spherical head diffraction model. An advantage of this model is that in the limit of an infinite source distance (plane wave incidence) the ITD and ILD depend only on the product of the frequency and head radius. Therefore, computations for a human listener at 500 Hz are the same as the computations at 1000 Hz for an animal with a head that is half the human size.

An initial comparison between ITD sensitivity and the iso-IPD boundaries offered little support for the EI. The brick-wall frequency of 1450 Hz is so high that many tones fall into the confusing region C where the IPD is greater than 180°. Tones with azimuths as small as 35° could be confusing like that, and much of the region of greatest ITD sensitivity falls into IPD region C when the azimuth is greater than 55°. The EI could be rescued by assuming that the frequency limits of the binaural system were established when heads were only half the diameter of present day human heads.

### 6.2. Tones experiments

In addition to asking whether an ecological connection actually exists between the frequency dependence of ITD sensitivity and the size of the head, one can also ask whether it is reasonable *even to expect* such a connection to exist. In the context of this paper, the frequency dependence corresponds to steady-state sine tones, but the sounds that are relevant in nature rarely meet those criteria. Therefore, one can question the value of our measurements and discussion depending on sine tones. However, the tonotopic organization of the auditory system means that different frequency regions contribute individually to an overall percept, and it is not unreasonable to characterize the influences from the regions by their responses to sine tones. For instance, specific contributions attributable to individual tonal components were demonstrated in experiments by Dye (1990). Similarly, ILD and ITD weighting functions measured by Macpherson and Middlebrooks (2002) for lowpass and high-pass noise bands agreed with expectations based on sine tones. The use of sine tones in an ecological context can be justified by recognizing the significance of tonotopic regions and frequency limits for those regions.

A second objection to an ecological perspective based on sine tones comes from the importance of transient sounds, both in nature and in sound localization. Unlike the phase ambiguities that occur with periodic sounds, there is no physical ambiguity for transients whatever the ITD. *A priori*, there is no ecological reason for limiting the frequency range of ITD sensitivity if sound source location is determined by the interaural delays for transients. However, apparently the properties of the binaural system have not evolved to deal optimally with transient sounds. Although transients, as typified by clicks, contain timing information that spans the entire frequency range of hearing, most of that information appears to be wasted. Experiments with filtered clicks (Yost et al., 1971) show that the ITD information in clicks is not available above 1500 Hz—the same as for sine tones. Shepard and Shepard and Colburn (1976) found that ITD discrimination for clicks is not better than for 500-Hz sine tones. Klumpp and Eady (1956) studied ITD discrimination for tones, noise bursts, and clicks and found that discrimination was worst for clicks. Hartmann and Rakerd (1989) showed that the interaural parameters for a sine tone dominate a sharp onset transient for the tone unless room reflections cause the interaural parameters to be unreliable (Franssen effect). Therefore, although transient sounds would appear to provide useful, consistent information across the entire audible spectrum, they have evidently not guided the evolution of the human binaural system. In summary, despite the impoverished nature of sine-tone stimuli, it is necessary to take experiments using sine tones seriously in assessing the limitations of binaural hearing in the real world.

### 6.3. Other species

An ecological approach to binaural hearing would be incomplete without consideration of species other than our own. Other species raise several problems. First, relating ITDs to azimuths using the SHM is less justifiable. The SHM, and its Woodworth model limit, assume a perfect sphere with featureless ears at antipodes on the equator. These four assumptions are approximately realized for human heads. They are not realized for most of the several dozen mammals for which ITDs have been measured and compared with anatomy where the ears are on the top of the head. For such animals, interaural properties depend on details of the pinnae much more than for humans. Tollin and Koka (2009) noted that the height of the pinnae in cat is almost equal to the head diameter. Koka et al. (2008) found that the pinnae make a significant contribution to ILD, at 10 kHz, but pinnae are not important for humans at the anatomically scaled frequency of 2 kHz. The ITDs measured on adult chinchilla by Lupo et al. (2011) were a factor of 2 larger than predicted by the SHM. Although the ears of the marmoset are not on top of the head, they are much larger compared to head size than for human (Slee and Young, 2010).

Beyond such technical matters, a comparable approach to other animals would require comparing available ITDs or head size to binaural perception. Animal perception can be inferred from behavioral experiments, especially sound localization tasks, but mere localization is not enough. It is also necessary to know that the localization is mediated by ITD in order to arrive at comparisons equivalent to our human study.

By observing structure in the frequency dependence of the localization performance of chinchillas, Heffner et al. (1994) inferred a frequency of 2.8 kHz for the upper limit of ITD utility. This frequency leads to an IPD of 180° when the ITD is about 180 μs. This ITD can be translated into azimuth given the plot for the adult chinchilla by Jones et al. (2011). Altogether, the data indicate that sources with azimuths greater than 60° will produce IPDs greater than 180°, and thus in confusing region C. Therefore, chinchillas can be expected to face the same ITD confusions as human listeners. However, Jones et al. also note that infant chinchillas have heads that are smaller by 50%, and Tollin and Koka (2009) found the same for cats. As for humans, such a reduction in head size causes all available ITDs to fall into useful IPD regions, and the large-IPD problem goes away.

A remarkable graph in a chapter by Heffner and Heffner (2003) shows a plot of the highest frequency at which binaural phase sensitivity has been observed against the maximum ITD allowed by the anatomy. The plot shows 12 animals including human. The plot has a strong negative slope—the larger the maximum available ITD, the lower the frequency limit for useable ITD. Drawing a line on this plot corresponding to an IPD of 180°, shows that with only two exceptions, all the animals are sensitive to frequencies and ITDs such that the IPD exceeds 180° (region C). The two exceptions are for the smallest animals, least weasel and kangaroo rat.

Tollin and Koka (2009) have noted that for cats, chinchillas, and humans the head diameter increases by about a factor of two from infancy (or the onset of hearing) to adulthood. Assuming that this rule applies to all the animals on the plot one can replot the points corresponding to available ITDs that are reduced by 50%. Then all the remaining 10 animals, except for two, experience only IPDs in the useful regions A and B. The exceptions are the horse and the domestic pig. Included with humans in the region where a 50% reduction in head size eliminates confusion, are Jamaican and Egyptian fruit bats, chinchilla, cat, Japanese and pig-tailed macaques, horse, and cow. Therefore, the observed binaural sensitivity appears to be appropriate for most of the animals in infancy and not in adulthood.

## 7. Conclusion

Ultimately, the calculations and measurements in this article have not solved the problem posed by the disconnect between the brick wall, where human sensitivity to ITD fine structure vanishes, and current human head sizes. They have brought greater quantitative precision to the discussion. The ecological interpretation, which attributes the vanishing of ITD sensitivity to head size was shown to fail unless the frequency limits of the brainstem evolved when the head was considerably smaller than current adult human heads. Alternatively, the small head hypothesis may apply to infancy and development. If the limits of binaural processing in the brainstem were fixed during infancy, the ecological interpretation of ITD sensitivity would again be supported. Although plasticity experiments suggest that the brainstem might easily have evolved or developed to accommodate a larger head size, it is possible that there was and is no pressing need for such a change because the problem posed by the disconnect could be solved at a higher level where ITD and ILD cues are combined. The ability of higher levels to switch between several spatial maps in real time given changing circumstances, even in ferrets (Keating et al., 2013), indicates a plasticity that relieves lower levels from the need to adapt.

### Conflict of interest statement

The authors declare that the research was conducted in the absence of any commercial or financial relationships that could be construed as a potential conflict of interest.

## Figures and Tables

**Figure A1:**
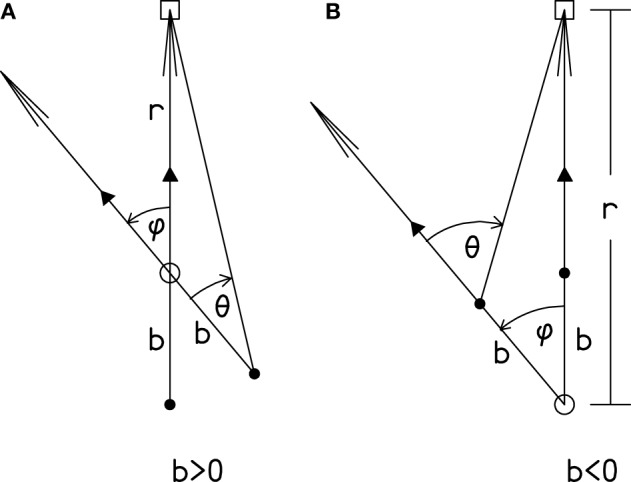
**The source of sound, indicated by the square, is fixed in space**. The head is shown in two orientations, defined by the arrows indicating the forward directions. Consistent with the definition of the forward direction, the arrow passes through the nose (triangle) and the COH (black dot). Because of the colinear assumption, it also passes through the axis of rotation shown by the open circle. In case **(A)** the center of the head is behind the axis of rotation so that *b* and ρ are positive. In case **(B)** the center of the head is in front of the axis of rotation so that *b* and ρ are negative. Equation (1) and the three steps apply to both cases. The directed arcs show the positive directions for θ and ϕ.

**Figure A2:**
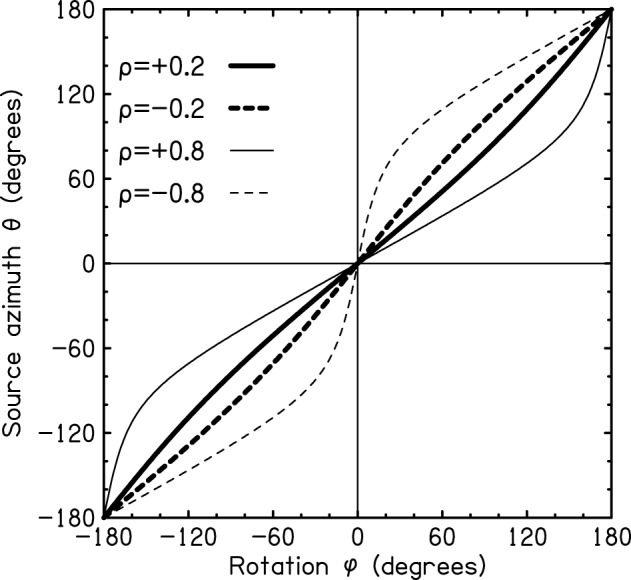
**Example calculation of the azimuth as a function of the head rotation angle for ρ = ±0.2 (heavy line) and ρ = ±0.8 (light line) for all possible values of the rotation**.

## Appendix

### Rotation-azimuth transform

The azimuth of a source with respect to an observer is an angle in the horizontal plane, as viewed from overhead. It is measured clockwise from the forward direction (determined by the nose) and extends through a full 360°, −180° to +180°. The azimuth angle occurs at the intersection of a line in the forward direction and a line that includes the center of the head (COH) and the source. The azimuth can be increased, for example by 30°, by moving the source location clockwise by 30° along a circle centered on the COH. Alternatively, the azimuth can be increased by 30° by leaving the source location fixed and rotating the head counterclockwise. However, this counterclockwise rotation of the head is not a rotation of 30°. That is because the axis of rotation for a human head, attached in the usual way to the human neck, does not pass through the COH. The purpose of this section is to show how to compensate for a discrepancy such as this. It develops the rotation-azimuth transformation.

The critical assumptions made in this treatment are (1) that the axis of rotation is vertical (perpendicular to the horizontal plane of the sources) and (2) that the extended line from the nose to the COH intersects the axis of rotation. The latter assumption is the “colinear assumption.”

#### Summary

The essential geometry is shown in Figure A1. The source is initially in the forward direction. The rotation of the head from the forward direction is angle ϕ. The resulting source azimuth is θ. The relationship between ϕ and θ depends on *b*, the distance from the axis of rotation to the COH, and it depends on *r*, the distance from the axis of rotation to the source. It does not depend on *b* and *r* separately, but only on the ratio, ρ = *b/r*, where ρ must be less than 1. There is a three step process for determining θ from ϕ: (1) Compute θ as

(1) $$\theta =\arctan\left[\frac{\sin\phi }{\rho +\cos\phi }\right].$$

Because *r* is positive, ratio ρ has the same sign as directed distance *b*. If the axis of rotation lies between the COH and the nose (Figure A1A), then *b* is positive, and the magnitude of θ is less than the magnitude of ϕ. If the COH lies between the axis of rotation and the nose (Figure A1B) then *b* is negative, and the magnitude of θ is greater than the magnitude of ϕ. Because sin ϕ/cos ϕ = tan ϕ, it is evident that in the limit of a very distant source (ρ = 0) Equation (1) leads to θ = ϕ.

(2) Realize that ϕ and θ must both have the same sign. If Equation (1) causes θ to have a sign opposite to ϕ then add 180° to the computed value of θ. This is the correct way to deal with the ambiguity caused by the principal value range of the arctangent.

(3) If θ turns out to be greater than 180°, bring θ into the range from −180° to +180° by subtracting 360°.

This three-step procedure is adequate for all possible rotations, positive and negative. Figure A2 shows the transformation between head rotation angle ϕ and the resulting source azimuth θ for two values of ρ, 0.2 and 0.8. The latter value corresponds to a source that is very close to the head, but it is included here because it illustrates mathematical asymmetries in the transformation that are not so apparent for small values of ρ such as 0.2.

#### Details of the transformation

All angles are measured from the forward direction. The forward direction is the directed line from the COH to the nose. The source azimuth θ is positive clockwise (as seen from the top) so that sources with positive azimuth are to the right of the observer. Consistent with this convention, the convention for the sign of the head rotation ϕ is positive counterclockwise—again putting a source to the right of the observer.

We define the COH as a point in the real head chosen so that the diffraction around the head is best approximated by the diffraction by a sphere centered on that point. The COH does not depend on the location of the ears. In general, a line drawn between the ears (the interaural axis) will not necessarily pass through the COH.

Equation (1) for azimuth θ comes from solving the triangle shown in Figure A1 using the sine law so that

(2) $$\frac{\sin\theta }{r}=\frac{\sin\text{​}\left(\theta -\phi \right)}{b}.$$

The arctangent formula is a simplification of this result from the sine law.
